# Gut microbiota’s influence on erysipelas: evidence from a two-sample Mendelian randomization analysis

**DOI:** 10.3389/fcimb.2024.1371591

**Published:** 2024-04-04

**Authors:** Lijie Bao, Zehui Wang, Lidong Wu, Zhiqiang Luo, Yibing Wang

**Affiliations:** Department of Emergency, The Second Affiliated Hospital, Jiangxi Medical College, Nanchang University, Nanchang, Jiangxi, China

**Keywords:** Mendelian randomization, erysipelas, gut microbiota, causal inference, genetics

## Abstract

**Background:**

Previous studies have suggested a link between gut microbiota and skin diseases, including erysipelas, an inflammatory skin condition. Despite this, the precise nature of the relationship between erysipelas and gut microbiota remains unclear and subject to debate.

**Methods:**

We conducted a Mendelian Randomization (MR) analysis using publicly available summary data from genome-wide association studies (GWAS) to explore the potential causal relationship between gut microbiota and erysipelas. Instrumental variables (IVs) were identified using a comprehensive set of screening methods. We then performed MR analyses primarily using the Inverse Variance Weighted (IVW) method, complemented by alternative approaches such as MR Egger, weighted median, simple mode, and weighted mode. A series of sensitivity analyses, including Cochran’s Q test, MR-Egger intercept test, Mendelian Randomization Pleiotropy RESidual Sum and Outlier (MR-PRESSO) test, and a leave-one-out test, were executed to ensure the robustness and validity of our findings.

**Results:**

We identified potential associations between erysipelas and various gut microbiota, including *Alcaligenaceae* (OR 1.23; 95% CI 1.06-1.43; p=0.006), *Rikenellaceae* (OR 0.77; 95% CI 0.67-0.90; p=0.001), and others. Notably, associations with *Actinomyces*, *Lachnospiraceae NC2004 group*, *Ruminiclostridium 9*, *Ruminococcaceae UCG014*, *Odoribacter*, and *Actinobacteria* were also observed. Sensitivity analyses confirmed the robustness of these associations.

**Conclusion:**

Our MR analysis suggests both potentially beneficial and harmful causal relationships between various gut microbiota and the incidence of erysipelas. This study provides new theoretical and empirical insights into the pathogenesis of erysipelas and underscores the potential for innovative preventive and therapeutic approaches.

## 1 Background

Erysipelas is a specific skin condition that primarily affects the superficial layer of the skin, leading to significant inflammation of the lymphatic vessels (Bisno and Stevens, 1996). Characterized by distinct, raised, erythematous patches, it stands out against the surrounding healthy skin (Swartz, 2004; Raff and Kroshinsky, 2016). Typically manifesting in the lower limbs and facial areas (Tartaglia, 2022) (Tartaglia, 2022), erysipelas is mainly caused by Group A Streptococcus, entering the body through minor injuries to the skin or mucous membranes. Occasionally, other types of Streptococci (such as groups B, C, G) or Staphylococcus aureus may also be involved (Dalal et al., 2017). Traditionally, erysipelas has been treated with penicillin antibiotics targeting streptococci. However, the increasing resistance to β-lactam antibiotics necessitates a reevaluation of treatment strategies (NICE, 2019; D. Yu et al., 2023). Clinical observations have noted a tendency for erysipelas to occur in individuals with weakened immune systems, prompting a reexamination of the pathogenesis of erysipelas from the perspective of gut microbiota.

The gut microbiota, a complex assembly of microorganisms in the human gastrointestinal tract, includes a variety of bacteria, fungi, viruses, and other organisms, profoundly impacting human health. These microorganisms play crucial roles in enhancing immune responses, facilitating digestion and metabolism, and influencing insulin secretion and resistance (Backhed et al., 2005; Gomaa, 2020). Recent studies have revealed a close link between gut microbiota and skin health, especially under the guidance of the “gut-skin axis” theory, further elucidating the connection between gut microbiota and skin diseases (De Pessemier et al., 2021; Stec et al., 2023). This has opened new avenues for exploring potential treatment strategies for erysipelas from the perspective of gut microbiota.

Against this backdrop of newfound understanding, the Mendelian randomization (MR) approach plays a pivotal role in our research. MR uses genetic variations as instrumental variables to establish causal relationships between environmental exposures and health outcomes, effectively reducing confounding factors and reverse causation compared to traditional observational studies. The random distribution of single nucleotide polymorphisms (SNPs) endows MR with a rigor akin to randomized controlled trials. Our study employs this method, integrating data from genome-wide association studies (GWAS), to investigate the potential causal relationship between gut microbiota and the risk of erysipelas. This research not only deepens our understanding of the pathophysiological mechanisms of erysipelas but also provides new theoretical and practical perspectives for developing prevention and treatment strategies.

## 2 Methods

### 2.1 Study design and data sources

**Figure 1:**
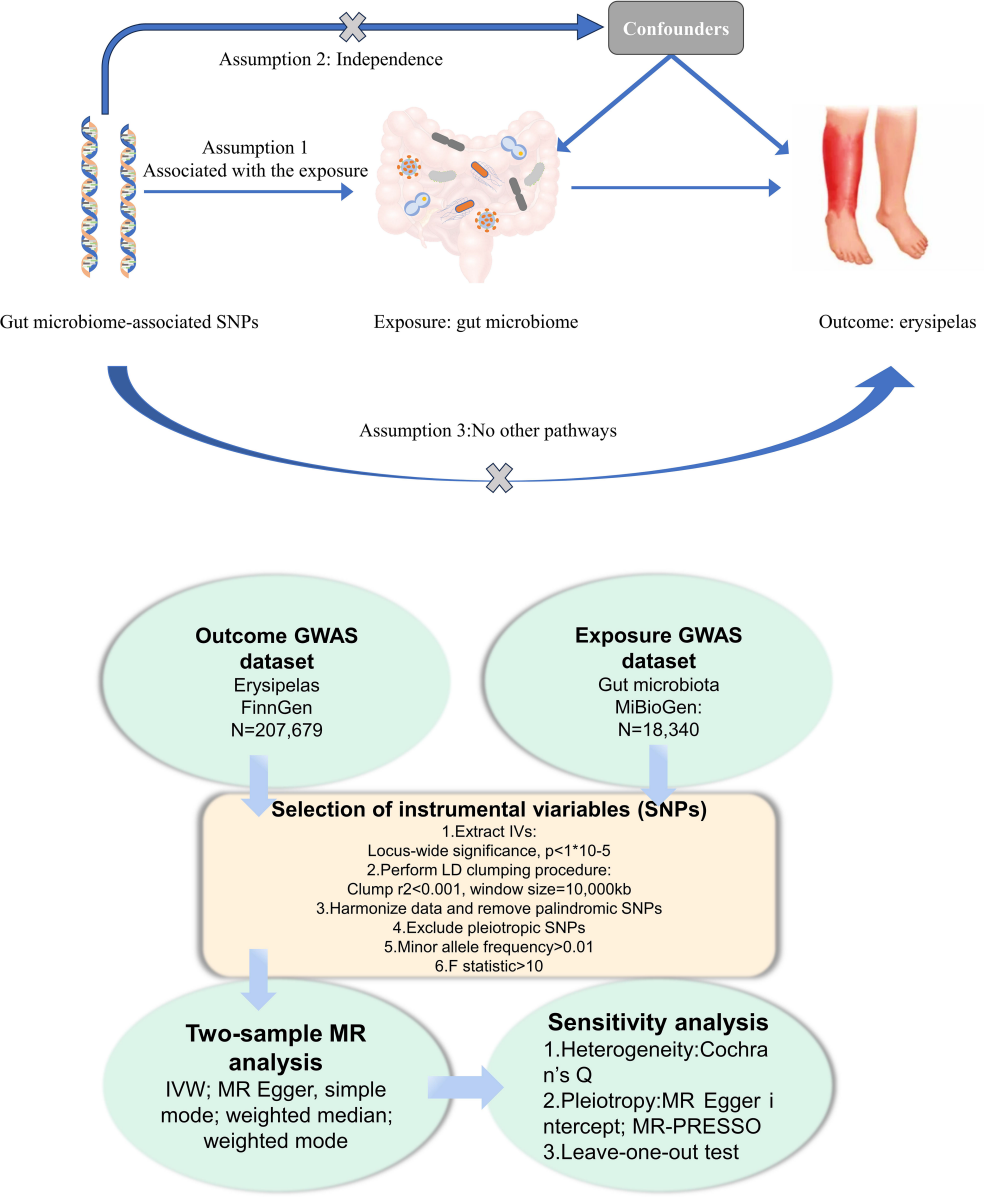
Flowchart of the present MR study and major assumptions. MR, Mendelian randomization; GWAS,genome-wide association study; SNPs, single nucleotide polymorphisms; IVW, inverse-variance weighted;LD, linkage disequilibrium; MR-PRESSO, MR pleiotropy residual sum and out.

In this study, we conducted a Mendelian Randomization (MR) analysis to investigate the causal links between gut microbiota and erysipelas. The overall workflow of our research is illustrated in **Figure 1**. Our approach began with the identification of genetic variants associated with the exposure, for which we extracted data from Genome-Wide Association Study (GWAS) summary statistics. These variants were then employed as instrumental variables (IVs) in our analysis. We executed a sequential two-sample MR analysis, incorporating five distinct MR methods to ensure robustness and reliability of our findings. To validate the significance of our associations, we undertook a comprehensive suite of sensitivity analyses. This included tests for heterogeneity and pleiotropy, as well as a leave-one-out analysis, thereby providing a thorough evaluation of the results and ensuring the validity of our conclusions.

Summary-level genomic data pertaining to gut microbiota were obtained from the MiBioGen study, which represents the most extensive and diverse genome-wide meta-analysis of gut microbiota conducted to date (Kurilshikov et al., 2021; Mibiogen, 2023). The study incorporates genome-wide genotyping data and 16S fecal microbiota profiles from 24 distinct cohorts, totaling 18,340 individuals. The majority of the study’s participants were of European ancestry, numbering 13,266. Microbial composition was profiled through targeted sequencing of the V4, V3-V4, and V1-V2 regions of the 16S rRNA gene. Taxonomic classification of the microbiota was conducted using direct taxonomic binning. After processing the 16S microbiome data, a total of 211 taxa were identified. This extensive array included 131 genera, 35 families, 20 orders, 16 classes, and 9 phyla. Detailed insights into the microbiota dataset and its comprehensive analysis are available in the original MiBioGen study publication (Kurilshikov et al., 2021).

For our study, the summary GWAS data for erysipelas were sourced from FinnGen, encompassing a cohort of 10,019 erysipelas patients and 197,660 controls. This dataset includes a comprehensive total of 16,380,453 SNPs, with all participants being of European ancestry (Kurki et al., 2023). To acquire the most relevant and extensive data, we conducted a meticulous search on the ‘ieu open gwas project’ website, using ‘erysipelas’ as the keyword. After a thorough review of the available datasets, we selected the most current and largest dataset, named ‘Erysipelas (Dataset: finn-b-AB1_ERYSIPELAS)’. This dataset stands out due to its extensive range of erysipelas-related data, offering a rich and diverse foundation for our analysis and significantly augmenting the depth of our research (Ben et al., 2020; Bristol, 2023).

### 2.2 Instrumental variables selection

To ascertain the accuracy and robustness of our findings on the causal relationship between gut microbiota and erysipelas risk, we employed a comprehensive series of quality control measures for filtering instrumental variables (IVs). Initially, we identified single-nucleotide polymorphisms (SNPs) with significant associations to the gut microbiome to serve as IVs. We included a set of SNPs demonstrating locus-wide significance levels below 1×10-5, thereby enriching the explained phenotypic variability. Additionally, to maintain the independence of IVs and reduce linkage disequilibrium effects, which could violate the principle of random allele assignment, we applied a clumping procedure with parameters set to r2 <0.001 and a distance of 10,000kb. In cases where exposure-related SNPs were absent in the outcome GWAS, we sought highly correlated proxy SNPs (r2 >0.8) via the SNiPA website (Arnold et al., 2015), although this was not necessary for our study. Palindromic SNPs and those with incompatible alleles were excluded to ensure the integrity of the MR analysis. Furthermore, to adhere to MR’s key assumption of independence from confounders, we manually screened and excluded SNPs significantly associated ((p<5×10-5) with potential confounders, as identified using the PhenoScanner GWAS database (Staley et al., 2016; Kamat et al., 2019). No SNPs associated with significant confounding factors were found. We also imposed a minimum minor allele frequency threshold of 0.01. Finally, to address weak instrumental variable bias, we calculated the F-statistic for each SNP (Burgess and Thompson, 2011), excluding any with an F-statistic below ten. The F-statistic is defined as R2(n-k-1)/k(1-R2), where n is the sample size, k represents the number of IVs, and R2 denotes the variance explained by the IVs.

### 2.3 Effect size estimate

In this study, we conducted a two-sample Mendelian Randomization (MR) analysis to investigate the causal relationship between characteristics of the gut microbiome and the risk of erysipelas. For gut microbiota features represented by multiple instrumental variables (IVs), we primarily employed the inverse-variance weighted (IVW) test, augmented by additional methodologies including MR-Egger, simple mode, weighted median, and weighted mode (Burgess et al., 2013). The IVW meta-analysis approach transforms the outcome effects of IVs on exposure into a weighted regression model, where the intercept is set to zero. This method, in the absence of horizontal pleiotropy, provides unbiased estimates by counteracting the effects of confounding variables (Holmes et al., 2017). However, it’s important to note that the MR-Egger method might be affected by outlier genetic variants, which could lead to imprecise estimations. Nonetheless, MR-Egger is capable of yielding unbiased estimates even when all selected IVs are invalid (Bowden et al., 2016b). The simple mode approach, while less powerful statistically compared to IVW, offers enhanced robustness against pleiotropy effects (Milne et al., 2017). The weighted median technique can deliver accurate and reliable effect estimates when at least 50% of the data come from valid instruments (Bowden et al., 2016a). Finally, in cases where genetic variants contravene the pleiotropy assumption, the weighted mode method proves beneficial (Hartwig et al., 2017).

### 2.4 Sensitivity analysis

To evaluate the potential effects of heterogeneity and pleiotropy among the instrumental variables (IVs) on our Mendelian Randomization (MR) findings, we conducted an extensive suite of sensitivity analyses. These analyses were pivotal in validating the robustness of our significant results. Heterogeneity among the genetic instruments was assessed using Cochran’s Q test and visually represented through funnel plots. Additionally, we rigorously examined potential horizontal pleiotropic effects of the IVs, utilizing both the MR Egger intercept and the Mendelian randomization pleiotropy residual sum and outlier (MR-PRESSO) global test. To further reinforce the accuracy of our causal effect estimates, we conducted a leave-one-out sensitivity analysis. This analysis was crucial in ensuring that our MR estimates were not disproportionately influenced by any single highly influential SNP. Moreover, the MR Steiger directionality test was implemented to deduce the direction of the causal relationship (Hemani et al., 2017). Causal inferences were deemed credible when the variance explained by the IVs on the exposure surpassed that on the outcome. All statistical procedures, encompassing both MR and sensitivity analyses, were performed using the ‘TwoSampleMR’ and ‘MRPRESSO’ packages in the R software environment (version 4.3.1), a robust and publicly accessible statistical platform. This article follows The STROBE-MR Statement (Skrivankova et al., 2021).

## 3 Results

Following our established criteria for instrumental variable (IV) selection, we meticulously identified 111 single nucleotide polymorphisms (SNPs) that demonstrated substantial associations with the gut microbiota. These SNPs, each surpassing the significance threshold of p<1×10-5, are indicative of notable relationships at various taxonomic levels of the gut microbiota, including family, genus, and phylum. Consequently, these SNPs were judiciously employed as instrumental variables in our analysis. For those interested in a deeper examination of these genetic markers, we have provided detailed information encompassing the effective alleles, alternative alleles, β values, standard errors (SEs), and p-values of these selected SNPs. This data can be found in the supplementary materials of our study (refer to **Supplementary Table 1**). We have shown in **Supplementary Table 2** which SNP markers are associated with which organism in the gut microbiota. The correlation between SNP and the risk of erysipelas in gut microbiome studies can be found in **Supplementary Figure 5**.

**Figure 2:**
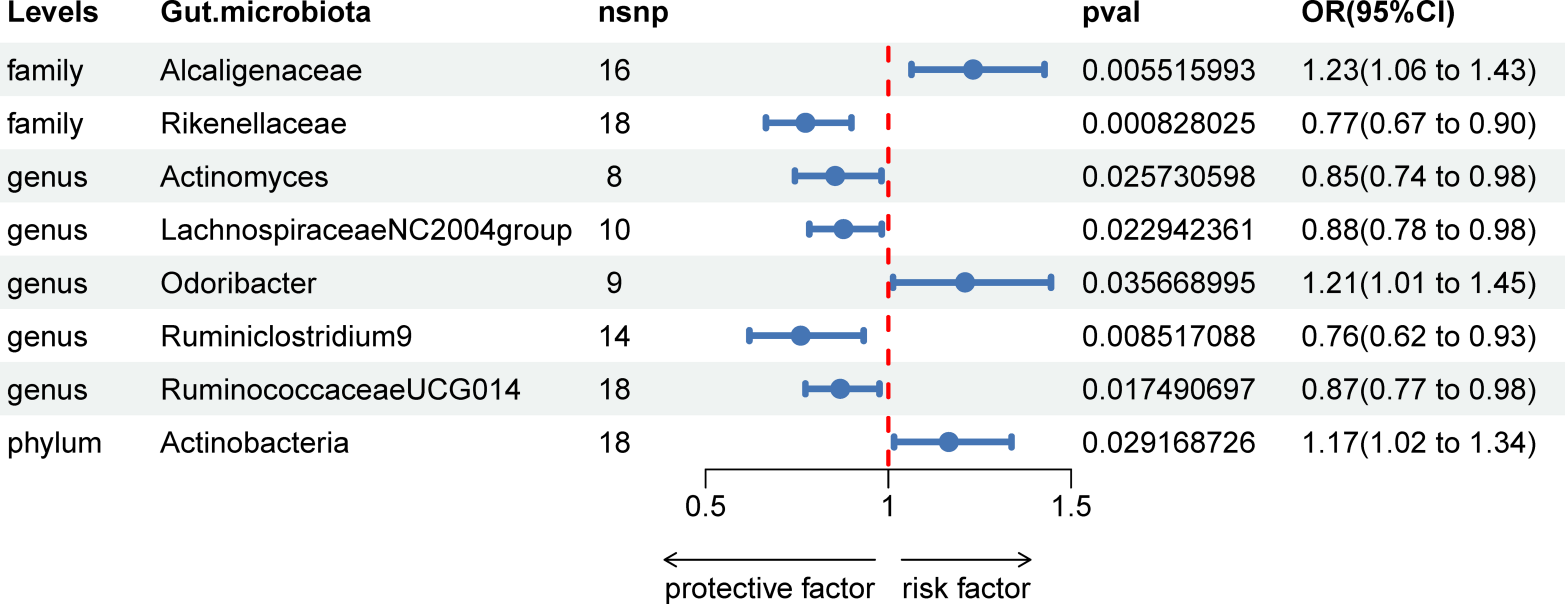
Associations of genetically predicted gut microbiota with erysipelas risk using IVW method SNPs, single nucleotide polymorphisms; OR, odds ratio; CI, confidence interval.

In our investigation, we carried out Mendelian Randomization (MR) analyses to assess potential causal links between various gut microbiota (exposure) and erysipelas (outcome). These analyses incorporated five distinct methodologies: Inverse Variance Weighted (IVW), MR Egger, weighted median, simple mode, and weighted mode. Using the IVW method, we discerned 8 gut bacteria taxa that exhibited potential causal relationships with erysipelas. We employed odds ratios (ORs) to articulate the association between increased abundance of these gut bacteria and the risk of erysipelas. Our IVW analysis revealed several key associations:①At the family taxonomic level, a surge in *Alcaligenes* abundance (OR 1.23; 95% CI 1.06-1.43; p=0.006) emerged as a risk factor for erysipelas. Conversely, *Rikenellaceae* showed a protective effect (OR 0.77; 95% CI 0.67-0.90; p=0.001).②At the genus level, protective factors against erysipelas included *Actinomyces* (OR 0.85; 95% CI 0.74-0.98; p=0.026), *Lachnospiraceae* NC2004 group (OR 0.88; 95% CI 0.78-0.98; p=0.023), Ruminiclostrinium 9 (OR 0.76; 95% CI 0.62-0.93; p=0.009), and *Ruminococcaceae UCG014* (OR 0.87; 95% CI 0.77-0.98; p=0.017). In contrast, *Odoribactor* was identified as a risk factor (OR 1.21; 95% CI 1.01-1.45; p=0.037).③At the phylum level, an increased abundance of *Actinobacteria* (OR 1.17; 95% CI 1.02-1.34; p=0.029) was associated with a higher risk of erysipelas. These findings are comprehensively presented in **Figure 2** and **Table 1** of our study.

**Table 1:** MR estimates for the association between gut microbiota and erysipelas (p<1×10-5).

Level	Microbiota	nsnp	Methods	Beta	OR (95% CI)	p value
**family**	Alcaligenaceae	16	MR Egger	0.21	1.24(0.66,2.31)	0.51
**family**	Alcaligenaceae	16	Weighted median	0.16	1.17(0.96,1.43)	0.12
**family**	Alcaligenaceae	16	Inverse variance weighted	0.21	1.23(1.06,1.43)	0.01
**family**	Alcaligenaceae	16	Simple mode	0.13	1.14(0.79,1.64)	0.51
**family**	Alcaligenaceae	16	Weighted mode	0.13	1.14(0.79,1.64)	0.50
**family**	Rikenellaceae	18	MR Egger	-0.25	0.78(0.48,1.25)	0.32
**family**	Rikenellaceae	18	Weighted median	-0.16	0.85(0.70,1.05)	0.13
**family**	Rikenellaceae	18	Inverse variance weighted	-0.26	0.77(0.67,0.90)	0.00
**family**	Rikenellaceae	18	Simple mode	-0.13	0.87(0.61,1.25)	0.48
**family**	Rikenellaceae	18	Weighted mode	-0.14	0.87(0.61,1.24)	0.45
**genus**	Actinomyces	8	MR Egger	-0.21	0.81(0.56,1.17)	0.30
**genus**	Actinomyces	8	Weighted median	-0.14	0.87(0.73,1.03)	0.11
**genus**	Actinomyces	8	Inverse variance weighted	-0.16	0.85(0.74,0.98)	0.03
**genus**	Actinomyces	8	Simple mode	-0.08	0.92(0.69,1.23)	0.59
**genus**	Actinomyces	8	Weighted mode	-0.17	0.85(0.65,1.11)	0.26
**genus**	LachnospiraceaeNC2004group	10	MR Egger	-0.01	0.99(0.62,1.58)	0.96
**genus**	LachnospiraceaeNC2004group	10	Weighted median	-0.14	0.87(0.75,1.01)	0.07
**genus**	LachnospiraceaeNC2004group	10	Inverse variance weighted	-0.13	0.88(0.78,0.98)	0.02
**genus**	LachnospiraceaeNC2004group	10	Simple mode	-0.19	0.82(0.64,1.06)	0.16
**genus**	LachnospiraceaeNC2004group	10	Weighted mode	-0.18	0.84(0.65,1.07)	0.19
**genus**	Odoribacter	9	MR Egger	0.54	1.72(0.95,3.11)	0.12
**genus**	Odoribacter	9	Weighted median	0.18	1.19(0.93,1.54)	0.17
**genus**	Odoribacter	9	Inverse variance weighted	0.19	1.21(1.01,1.45)	0.04
**genus**	Odoribacter	9	Simple mode	0.19	1.21(0.83,1.75)	0.35
**genus**	Odoribacter	9	Weighted mode	0.18	1.20(0.84,1.73)	0.35
**genus**	Ruminiclostridium9	14	MR Egger	0.41	1.51(0.55,4.14)	0.44
**genus**	Ruminiclostridium9	14	Weighted median	-0.31	0.73(0.58,0.93)	0.01
**genus**	Ruminiclostridium9	14	Inverse variance weighted	-0.27	0.76(0.62,0.93)	0.01
**genus**	Ruminiclostridium9	14	Simple mode	-0.34	0.72(0.47,1.10)	0.15
**genus**	Ruminiclostridium9	14	Weighted mode	-0.34	0.71(0.49,1.04)	0.10
**genus**	RuminococcaceaeUCG014	18	MR Egger	-0.26	0.77(0.57,1.05)	0.12
**genus**	RuminococcaceaeUCG014	18	Weighted median	-0.15	0.86(0.72,1.02)	0.08
**genus**	RuminococcaceaeUCG014	18	Inverse variance weighted	-0.14	0.87(0.77,0.98)	0.02
**genus**	RuminococcaceaeUCG014	18	Simple mode	-0.18	0.84(0.63,1.12)	0.26
**genus**	RuminococcaceaeUCG014	18	Weighted mode	-0.17	0.84(0.69,1.03)	0.12
**phylum**	Actinobacteria	18	MR Egger	-0.36	0.70(0.37,1.31)	0.28
**phylum**	Actinobacteria	18	Weighted median	0.09	1.10(0.89,1.35)	0.38
**phylum**	Actinobacteria	18	Inverse variance weighted	0.15	1.17(1.02,1.34)	0.03
**phylum**	Actinobacteria	18	Simple mode	0.46	1.59(1.07,2.34)	0.03
**phylum**	Actinobacteria	18	Weighted mode	-0.01	0.99(0.73,1.33)	0.93

⁎nsnp, the number of SNP.

The results from additional analytical approaches are meticulously detailed in **Table 1**. A scatter plot, as depicted in **Supplementary Figure 2**, provides a visual representation of potential causal relationships between the gut microbiota and erysipelas. In this plot, lines of different colors represent various Mendelian Randomization (MR) methodologies, including Inverse Variance Weighted (IVW), weighted median, MR-Egger, weighted mode, and simple mode. Each of these methods contributes to estimating the causal effects exerted by the gut microbiota on erysipelas. The slope value in these analyses, corresponding to the b value derived from the five methodologies, indicates the magnitude of the gut microbiota’s causal impact on erysipelas. A larger absolute value of the slope suggests a more pronounced causal effect. In this context, a positive slope implies that the exposure – the abundance of certain gut microbiota – acts as a risk factor for erysipelas. Conversely, a negative slope suggests a protective effect against erysipelas.

In the course of our Mendelian Randomization (MR) analysis, we have successfully pinpointed eight potential causal relationships between the gut microbiota and erysipelas. To ascertain the reliability and robustness of our findings, we conducted an extensive array of sensitivity analyses. These analyses were meticulously designed to assess the possible influences of heterogeneity and pleiotropy within our selected instrumental variables (IVs). This comprehensive approach was crucial in validating the integrity and accuracy of our results, ensuring that the identified associations were not artifacts of underlying variability or confounding influences among the IVs.

To explore the presence of potential heterogeneity among our selected instrumental variables (IVs), we conducted Cochran’s Q tests. The results of these tests were crucial, as all p-values were found to exceed the threshold of 0.05. This outcome indicates a lack of significant heterogeneity among the IVs, reinforcing the consistency of our findings. Additionally, we meticulously assessed the possibility of horizontal pleiotropy, a factor that could potentially skew our results. This assessment was performed using two robust tests: the MR-Egger intercept and the MR-PRESSO global test. Significantly, both tests yielded p-values greater than 0.05, suggesting the absence of notable horizontal pleiotropy in our analysis. These findings, which contribute to the validity of our results, are detailed in **Table 2**.

**Table 2:** Evaluation of heterogeneity and directional pleiotropy using different methods.

Level	Microbiota	Heterogeneity	Horizontal pleiotropy	
		Cochran’s Q p	MR-Egger intercept p	MR-PRESSO global test p
**family**	Alcaligenaceae	0.85	0.80	0.85
**family**	Rikenellaceae	0.24	0.19	0.27
**genus**	Actinomyces	0.38	0.29	0.43
**genus**	LachnospiraceaeNC2004group	0.62	0.55	0.65
**genus**	Odoribacter	0.64	0.72	0.65
**genus**	Ruminiclostridium9	0.06	0.09	0.08
**genus**	RuminococcaceaeUCG014	0.45	0.43	0.50
**phylum**	Actinobacteria	0.45	0.57	0.46

In our endeavor to reinforce the robustness of our findings, we undertook several additional analyses. These included the construction of forest plots and conducting leave-one-out analyses. The forest plots provided a visual representation of the individual effects of each SNP, while the leave-one-out analyses offered insights into the impact of each SNP on the overall Mendelian Randomization (MR) results. Collectively, these analyses revealed a crucial aspect: no single SNP exerted a disproportionate influence on the overall MR analysis. This observation is significant as it substantiates the resilience and stability of our findings. The detailed outcomes of these additional analyses are depicted in **Supplementary Figure 3**, which provides a comprehensive visual overview of the results.

## 4 Discussion

In our Mendelian Randomization (MR) study, we methodically explored the potential causal relationship between gut microbiota composition and erysipelas risk. Utilizing summary statistics from comprehensive genome-wide association studies (GWAS) on both gut microbiota and erysipelas, our detailed analysis identified eight specific bacterial taxa with potential causal links to erysipelas. Our findings suggest that an increased abundance of the bacterial families *Rikenellaceae*, *Actinomyces*, *Lachnospiraceae NC2004 group*, Ruminiclostridium 9, and *Ruminococcaceae UCG014* may act as protective factors against erysipelas. Conversely, an elevated presence of *Alcaliginaceae*, *Odoribacter*, and *Actinobacteria* appears to elevate the risk of developing erysipelas. This investigation illuminates the significant role that variations in gut microbiota diversity and abundance may play in the etiology of erysipelas, underscoring the intricate interplay between gut microbial composition and systemic health conditions.

In our Mendelian Randomization study, we observed that *LachnospiraceaeNC2004group*, *Ruminiclostridium9*, and *RuminococcaceaeUCG014*, all members of the class *Clostridia* within the *Firmicutes* phylum, are associated with a reduced risk of erysipelas. *Clostridia*, known for its anaerobic properties in the gut, plays a vital role in the digestion of cellulose and other complex carbohydrates, producing short-chain fatty acids (SCFAs) such as butyric acid, acetic acid, and propionic acid (Petersen et al., 2019). There is existing literature suggesting that SCFAs may exert anti-inflammatory effects on the skin (De Pessemier et al., 2021), proposing a mechanism by which Clostridia might influence the onset of skin diseases through SCFA production. Erysipelas, being an infectious skin disease, appears to be inversely related to the abundance of these Clostridia members in our findings. While this aligns with the current understanding of SCFA’s role in skin health, it remains to be determined whether the protective effect against erysipelas by *LachnospiraceaeNC2004group*, *Ruminiclostridium9*, and *RuminococcaceaeUCG014* is directly mediated through SCFA production. Further research is crucial to unravel the precise mechanisms underlying these associations.

In our study, we identified significant associations between several members of the *Bacteroidetes* phylum, namely *Rikenellaceae*, *Alcaligenaceae*, and *Odoribacter*, and the risk of erysipelas. *Rikenellaceae*, primarily found in the animal intestines, has been linked to intestinal health and inflammation (Zhang et al., 2020). Recent studies have proposed *Rikenellaceae* as a potential protective factor against psoriatic arthritis (N. Yu et al., 2023), and our findings similarly suggest its protective role in erysipelas. The correlation of *Rikenellaceae* with both psoriatic arthritis and erysipelas, both inflammatory diseases, underscores the significance of gut microbiota in human health. *Alcaligenaceae*, encompassing genera such as Alcaligenes, Pseudomonas, and Stenotrophomonas, has diverse implications. Certain species within this family, including Pseudomonas and Stenotrophomonas, are known to cause skin and soft tissue infections (Nagoba et al., 2017). Interestingly, our study observed an increased abundance of *Alcaligenaceae* correlating with a higher risk of erysipelas. This raises intriguing questions about the role of gut microbiota, as opposed to direct contact, in contributing to skin infections. Moreover, our analysis identified *Odoribacter* as a risk factor for erysipelas, a novel finding since previous research has not established this association. This insight positions our study at the forefront of exploring the relationship between *Odoribacter* and erysipelas, paving the way for future research to uncover the specific mechanisms at play.

In our investigation, we delved into the role of Actinomyces, a genus within the Actinobacteria phylum, which is a regular component of human and animal microbiota. Actinomyces are commonly found in the oral, digestive, and reproductive tracts, where they typically exist in a symbiotic relationship. Despite their generally benign presence, they can sometimes become pathogenic, particularly when they enter the body through oral or skin lesions, leading to chronic purulent inflammation known as actinomycosis (Urban and Gajdacs, 2021). Intriguingly, our study identified Actinobacteria as a risk factor for erysipelas. However, it also revealed that Actinomyces, a member of the Actinobacteria family, acts as a protective factor against erysipelas. This apparent contradiction presents a complex picture of Actinobacteria’s role in erysipelas. It suggests that while the phylum as a whole may increase the risk, specific genera like Actinomyces could have a protective effect. Such findings necessitate a cautious approach in interpreting the role of Actinobacteria in erysipelas. Further clinical research is imperative to unravel these complexities and delineate the precise contributions of various Actinobacteria members to the pathophysiology of erysipelas.

This study marks a pioneering effort in utilizing Mendelian Randomization (MR) analysis to explore the causal impact of gut microbiota on erysipelas. Diverging from traditional observational studies that often grapple with confounding factors and reverse causation, our MR-based approach delivers results with enhanced reliability. The discovery of specific bacterial taxa that exhibit causal relationships with erysipelas opens up unprecedented and valuable avenues for prevention and treatment strategies. These strategies, informed by the influence of gut microbiota, hold significant promise in revolutionizing our understanding and management of erysipelas.

The single nucleotide polymorphisms (SNPs) associated with gut microbiota, utilized in our study, are derived from the most expansive genome-wide association study (GWAS) meta-analysis conducted to date. This fact lends substantial credibility to the instrumental variables (IVs) used in our research. The considerable size of the sample pool, along with the implementation of various sensitivity analyses, significantly bolsters the reliability and validity of our findings.

However, it is important to acknowledge a potential limitation in our study’s demographic scope. The gut microbiota GWAS data predominantly represent individuals of European ancestry, with a relatively limited inclusion of data from non-European ancestries. Furthermore, the erysipelas GWAS data are exclusively composed of European ancestry individuals. This demographic concentration may introduce a degree of bias, potentially limiting the generalizability of our results to a wider population.

Our study lays the groundwork for a plausible causal link between gut microbiota and erysipelas, yet it is crucial to recognize the absence of direct mechanistic research to support these findings fully. This gap underscores the necessity for future investigations aimed at elucidating the specific mechanisms by which gut microbiota influences erysipelas. Such research, especially focusing on the 8 identified bacterial taxa, is pivotal for a more nuanced understanding of erysipelas’ etiology and for paving the way towards novel preventive and therapeutic approaches.

## 5 Conclusion

This groundbreaking study, utilizing Mendelian Randomization (MR) analysis, provides compelling genetic evidence for the causal role of gut microbiota in erysipelas. The identified gut microbiota, whether protective or harmful concerning erysipelas, potentially unveil new and invaluable pathways for erysipelas prevention and treatment. These findings suggest that interventions targeting gut microbiota composition could be pivotal in managing erysipelas, thereby offering innovative approaches to addressing this skin condition. Our research highlights the significant impact of gut microbiota on erysipelas, paving the way for future studies and clinical applications focused on microbiota-mediated strategies.

## Data availability statement

The original contributions presented in the study are included in the article/**Supplementary Material**. Further inquiries can be directed to the corresponding author.

## Author contributions

LB: Data curation, Writing – original draft, Writing – review & editing, Methodology. ZW: Data curation, Writing – original draft, Software, Visualization, Writing – review & editing. LW: Validation, Writing – original draft, Writing – review & editing. ZL: Writing – original draft. YW: Conceptualization, Data curation, Writing – original draft, Writing – review & editing.
