# Supplementary material for: Gut microbiota’s influence on erysipelas: evidence from a two-sample Mendelian randomization analysis
Source: Front Cell Infect Microbiol. 2024 Apr 4;14:1371591. doi: 10.3389/fcimb.2024.1371591 (PMC11024262; doi:10.3389/fcimb.2024.1371591)
Supplement: Supplementary file 1 [file DataSheet_1.pdf]

Supplementary Material

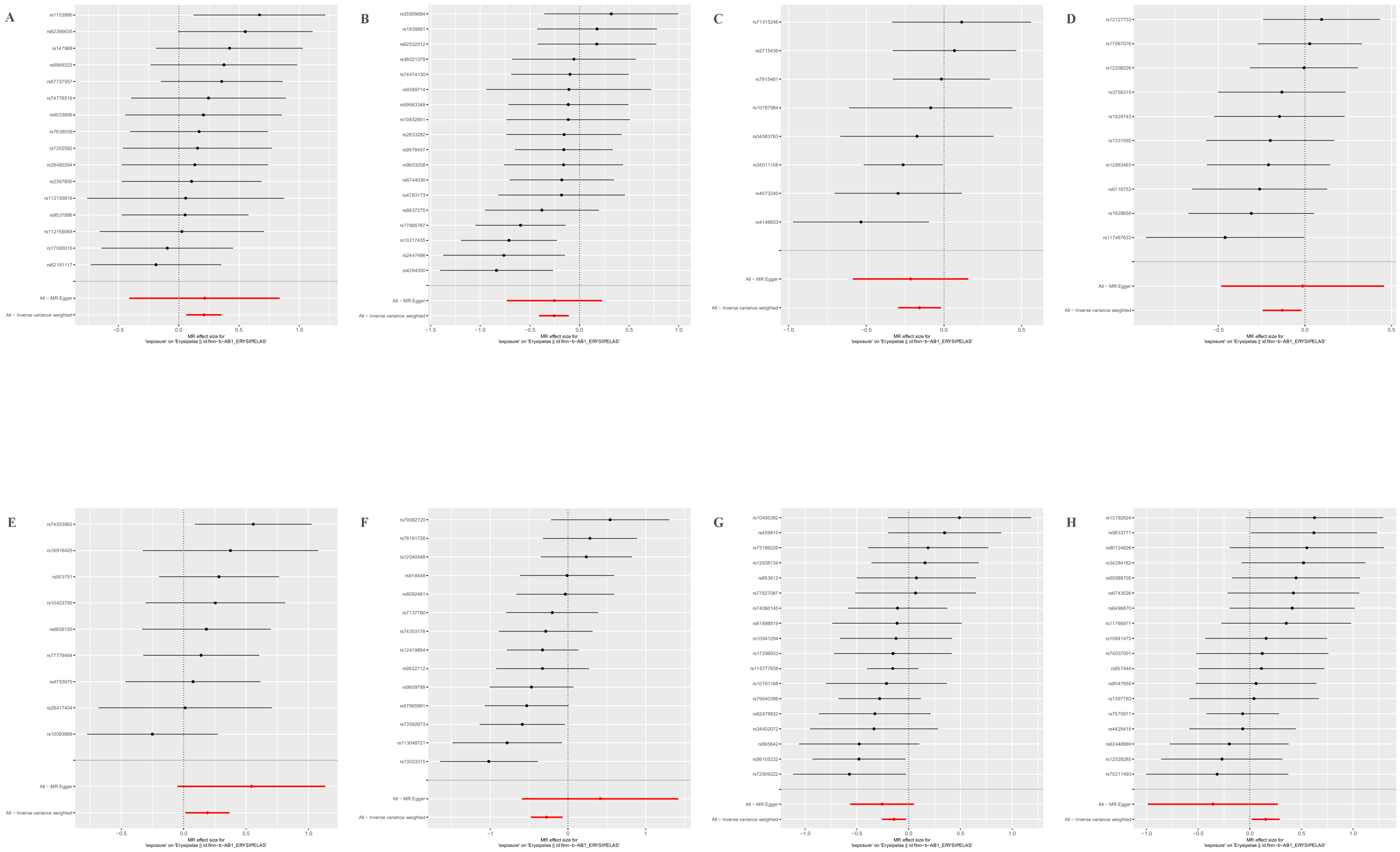

Supplementary Figure 1. Forest plots of the causal effects of gut microbiota on the risk of erysipelas. **(A)** Alcaligenaceae; **(B)** Rikenellaceae; **(C)** Actinomyces; **(D)** LachnospiraceaeNC2004group; **(E)** Odoribacter; **(F)** Ruminiclostridium9; **(G)** RuminococcaceaeUCG014; **(H)** Actinobacteria.

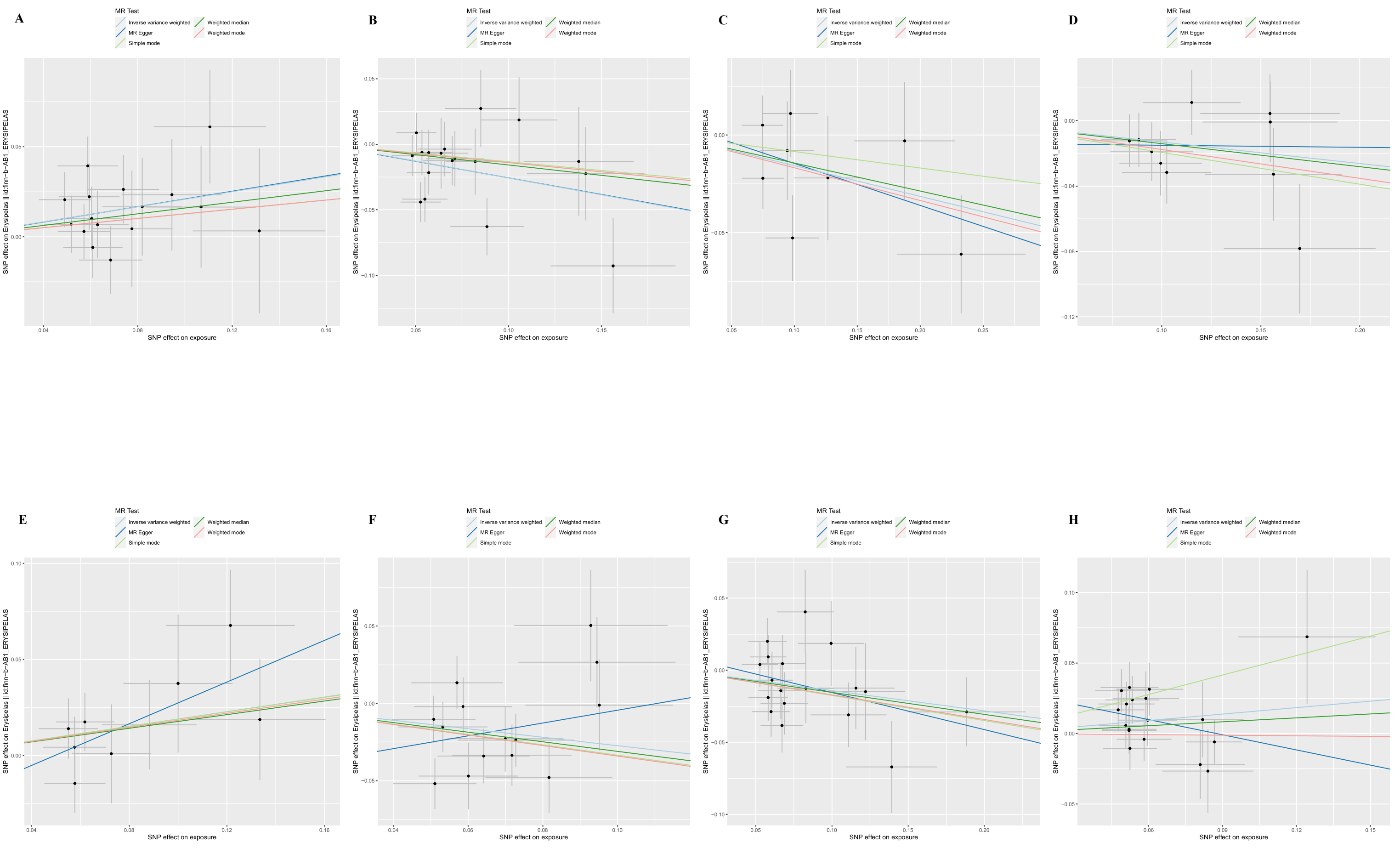

Supplementary Figure 2. Scatter plots of the causal effect of gut microbiota on the risk of erysipelas. **(A)** Alcaligenaceae; **(B)** Rikenellaceae; **(C)** Actinomyces; **(D)** LachnospiraceaeNC2004group; **(E)** Odoribacter; **(F)** Ruminiclostridium9; **(G)** RuminococcaceaeUCG014; **(H)** Actinobacteria.

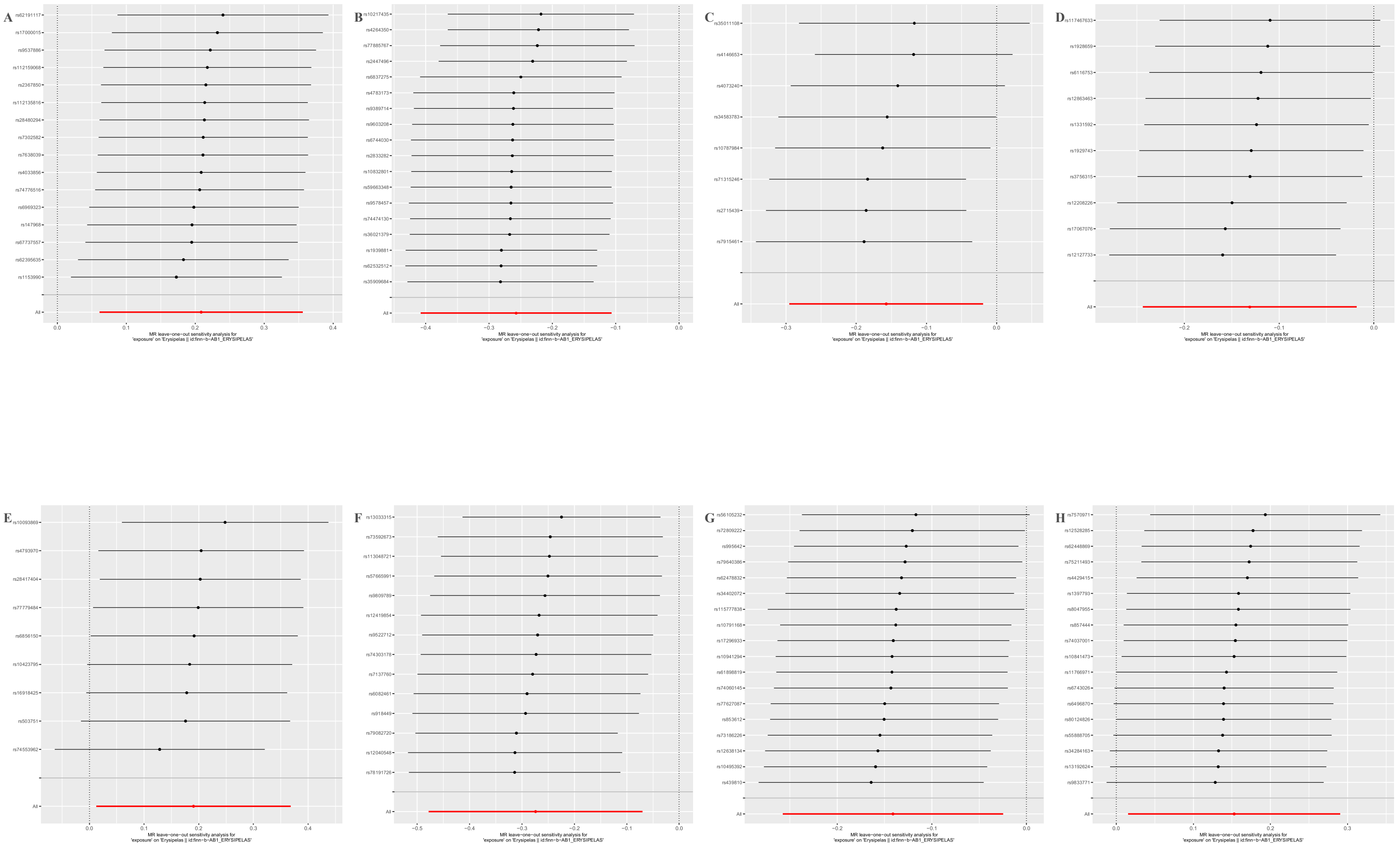

Supplementary Figure 3. Leave-one-out sensitivity analyses of the causal effects of gut microbiota on the risk of erysipelas. **(A)** Alcaligenaceae; **(B)** Rikenellaceae; **(C)** Actinomyces; **(D)** LachnospiraceaeNC2004group; **(E)** Odoribacter; **(F)** Ruminiclostridium9; **(G)** RuminococcaceaeUCG014; **(H)** Actinobacteria.

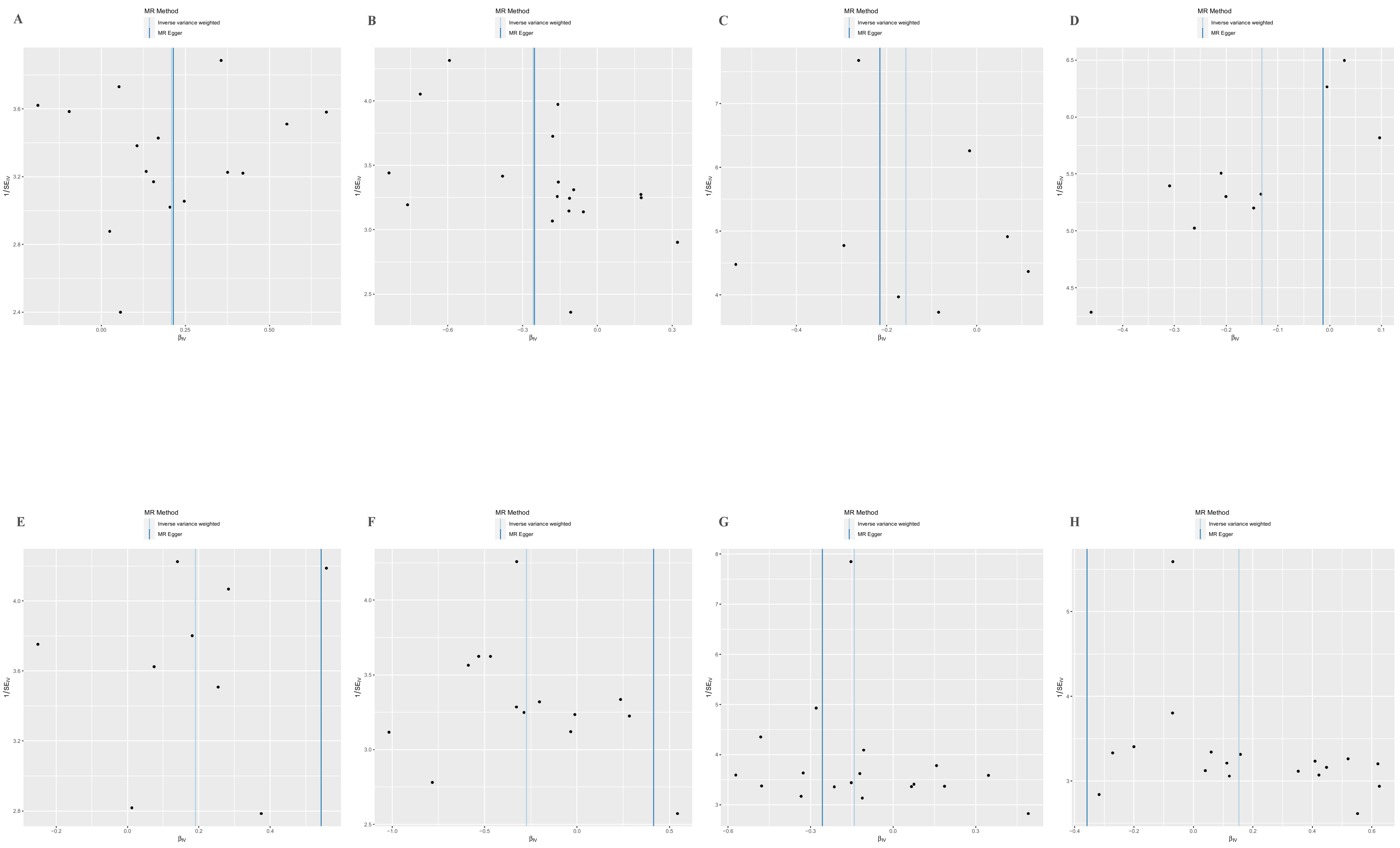

Supplementary Figure 4. Funnel-plot of the causal effects of gut microbiota on the risk of erysipelas. **(A)** Alcaligenaceae; **(B)** Rikenellaceae; **(C)** Actinomyces; **(D)** LachnospiraceaeNC2004group; **(E)** Odoribacter; **(F)** Ruminiclostridium9; **(G)** RuminococcaceaeUCG014; **(H)** Actinobacteria.

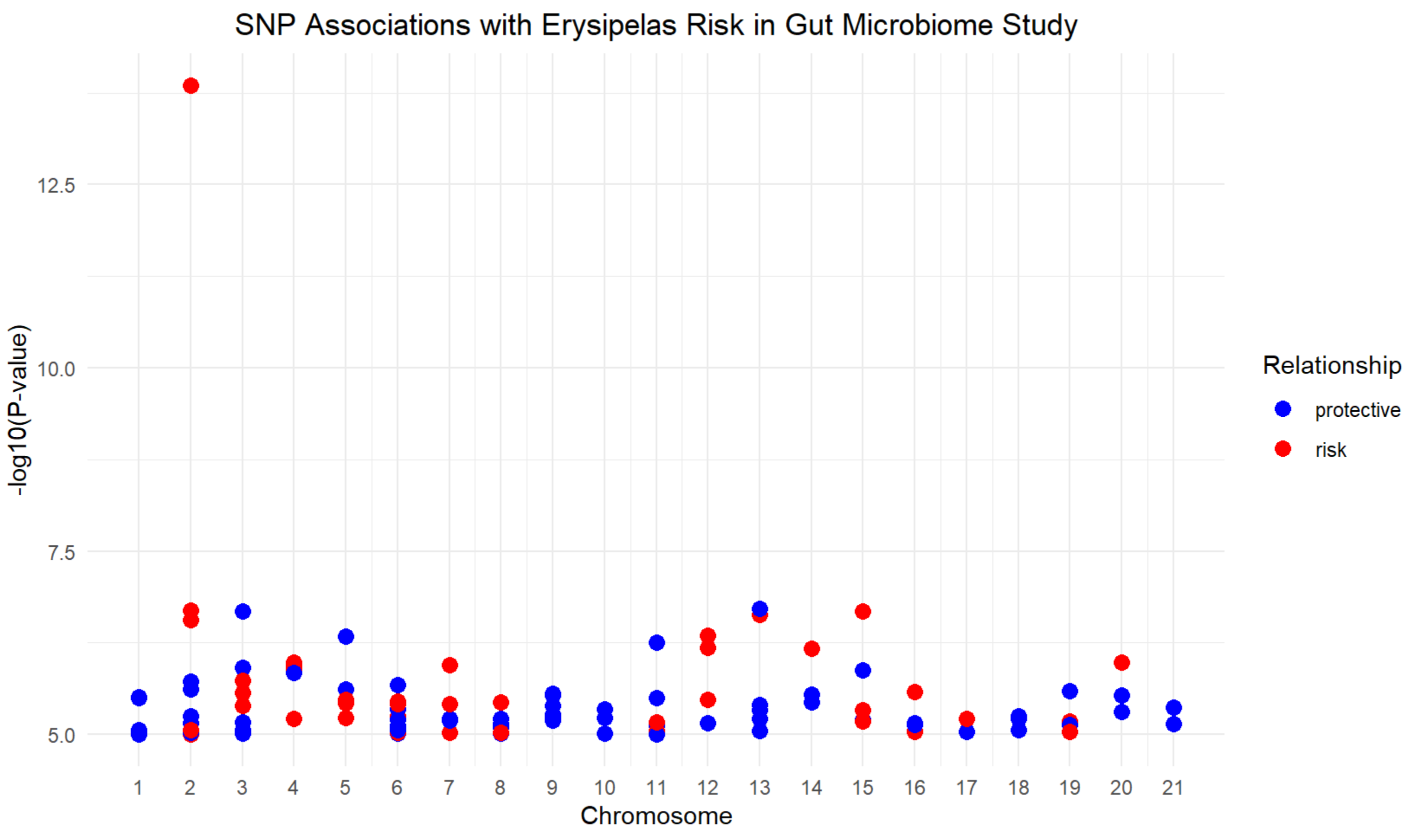

Supplementary Figure 5. SNP associations with Erysipelas risk in gut microbiome study.
